# High-grade astrocytoma with piloid features (HGAP): the Charité experience with a new central nervous system tumor entity

**DOI:** 10.1007/s11060-021-03749-z

**Published:** 2021-04-27

**Authors:** Katja Bender, Eilís Perez, Mihaela Chirica, Julia Onken, Johannes Kahn, Winfried Brenner, Felix Ehret, Philipp Euskirchen, Arend Koch, David Capper, David Kaul

**Affiliations:** 1 Charité - Universitätsmedizin Berlin, Corporate Member of Freie Universität Berlin and Humboldt-Universität zu Berlin, Department of Radiation Oncology, Charitéplatz 1, 10117 Berlin, Germany; 2 Charité - Universitätsmedizin Berlin, Corporate Member of Freie Universität Berlin and Humboldt-Universität zu Berlin, Department of Neuropathology, Charitéplatz 1, 10117 Berlin, Germany; 3 German Cancer Consortium (DKTK), Partner Site Berlin, German Cancer Research Center (DKFZ), Heidelberg, Germany; 4 Charité - Universitätsmedizin Berlin, Corporate Member of Freie Universität Berlin and Humboldt-Universität zu Berlin, Department of Neurosurgery, Charitéplatz 1, 10117 Berlin, Germany; 5 Charité - Universitätsmedizin Berlin, Corporate Member of Freie Universität Berlin and Humboldt-Universität zu Berlin, Department of Radiology, Charitéplatz 1, 10117 Berlin, Germany; 6 Charité - Universitätsmedizin Berlin, Corporate Member of Freie Universität Berlin and Humboldt-Universität zu Berlin, Department of Nuclear Medicine, Charitéplatz 1, 10117 Berlin, Germany; 7 Charité - Universitätsmedizin Berlin, Corporate Member of Freie Universität Berlin and Humboldt-Universität zu Berlin, Department of Neurology, Charitéplatz 1, 10117 Berlin, Germany

**Keywords:** High-grade astrocytoma with piloid features, HGAP, Anaplastic astrocytoma with piloid features, MC AAP, Methylation-based classification, Case series

## Abstract

**Purpose:**

High-grade astrocytoma with piloid features (HGAP) is a recently described brain tumor entity defined by a specific DNA methylation profile. HGAP has been proposed to be integrated in the upcoming World Health Organization classification of central nervous system tumors expected in 2021. In this series, we present the first single-center experience with this new entity.

**Methods:**

During 2017 and 2020, six HGAP were identified. Clinical course, surgical procedure, histopathology, genome-wide DNA methylation analysis, imaging, and adjuvant therapy were collected.

**Results:**

Tumors were localized in the brain stem (n = 1), cerebellar peduncle (n = 1), diencephalon (n = 1), mesencephalon (n = 1), cerebrum (n = 1) and the thoracic spinal cord (n = 2). The lesions typically presented as T1w hypo- to isointense and T2w hyperintense with inhomogeneous contrast enhancement on MRI. All patients underwent initial surgical intervention. Three patients received adjuvant radiochemotherapy, and one patient adjuvant radiotherapy alone. Four patients died of disease, with an overall survival of 1.8, 9.1, 14.8 and 18.1 months. One patient was alive at the time of last follow-up, 14.6 months after surgery, and one patient was lost to follow-up. Apart from one tumor, the lesions did not present with high grade histology, however patients showed poor clinical outcomes.

**Conclusions:**

Here, we provide detailed clinical, neuroradiological, histological, and molecular pathological information which might aid in clinical decision making until larger case series are published. With the exception of one case, the tumors did not present with high-grade histology but patients still showed short intervals between diagnosis and tumor progression or death even after extensive multimodal therapy.

## Introduction

Genome-wide DNA methylation profiling revealed a common DNA methylation profile that initially had been named *methylation-class anaplastic astrocytoma with piloid features* (MC AAP) but has recently been renamed to *high-grade astrocytoma with piloid features* (HGAP) [1]. This new tumor entity is expected to enter the upcoming WHO classification of CNS tumors of 2021. It will be among the first CNS tumor entities defined by a specific DNA methylation profile. Until surrogate markers are identified, DNA methylation profiling is required to establish the diagnosis of HGAP. Comprehensive clinical data on this new entity are extremely rare at this point. In this single-institution experience, we contribute six cases of HGAP, providing clinical, neuroradiological, histological and molecular pathological information as well as a precise description of our treatment approaches.

## Materials and methods

We included all patients diagnosed with HGAP between June 2017 and October 2020 at our institution. Tumors were identified by DNA methylation-based brain tumor classification [2]. DNA methylation array data were processed with the R/Bioconductor package minfi (version 1.36.0) as previously described [2, 3]. Dimensionality reduction for visualization was performed by computing a two-dimensional t-distributed stochastic neighbor embedding (t-SNE) via the R package Rtsne (version 0.15) using the 32,000 most variable CpG sites according to standard deviation [4, 5]. The following non-default parameters were used: theta = 0.5000 iterations, and a perplexity value of 18.

Clinical data were gathered from institutional medical records and included demographic information, histopathologic and immunohistochemical testing, neuroradiological imaging, treatment, and outcome data. Karnofsky performance status (KPS) was assessed postoperatively. Dates of death were obtained from the municipal death register. Progression-free survival (PFS) was determined as the time span between first surgical resection and radiological tumor recurrence. Overall survival (OS) was calculated from the day of the first surgery to the date of death.

## Results

### Patient characteristics

**Fig. 1:**
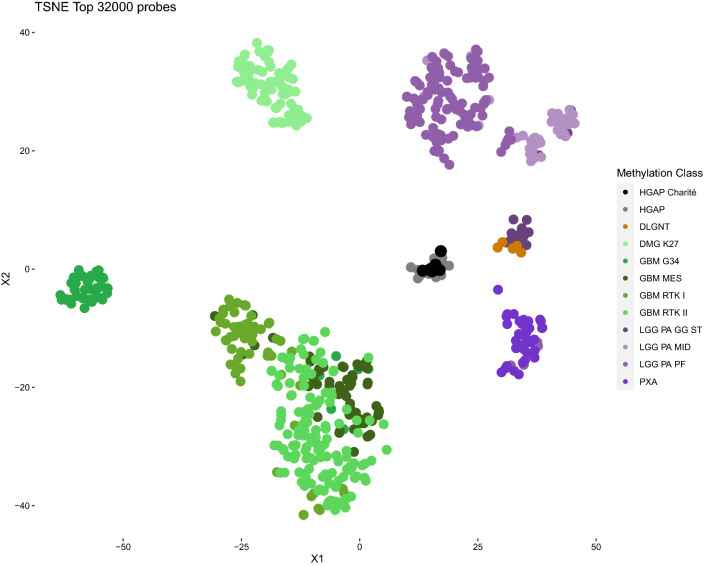
T-SNE analysis of DNA methylation data of the six high-grade astrocytomas with piloid features (HGAP) of this series together with a reference cohort of 11 different molecular tumor classes (n = 616) [2]. The six cases clearly group together with the reference cohort of HGAP. Reference methylation classes: *HGAP* High-grade astrocytoma with piloid features (16 cases); *DLGNT* diffuse leptomeningeal glioneuronal tumor (6 cases); *DMG K27* diffuse midline glioma, H3 K27M mutant (78 cases); *GBM G34* glioblastoma, IDH wild-type, H3 G34 mutant (41 cases); *GBM MES* glioblastoma, IDH wild-type, subclass mesenchymal (56 cases); *GBM RTK I* glioblastoma, IDH wild-type, subclass RTK I (64 cases); *GBM RTK II* glioblastoma IDH wild-type, subclass RTK II (138 cases); *LGG PA GG ST* low-grade glioma, subclass hemispheric pilocytic astrocytoma and ganglioglioma (24 cases); *LGG PA MID* low-grade glioma, subclass midline pilocytic astrocytoma (38 cases); *LGG PA PF* low-grade glioma, subclass posterior fossa pilocytic astrocytoma (114 cases); *PXA* (anaplastic) pleomorphic xanthoastrocytoma (35 cases)

**Fig. 2:**
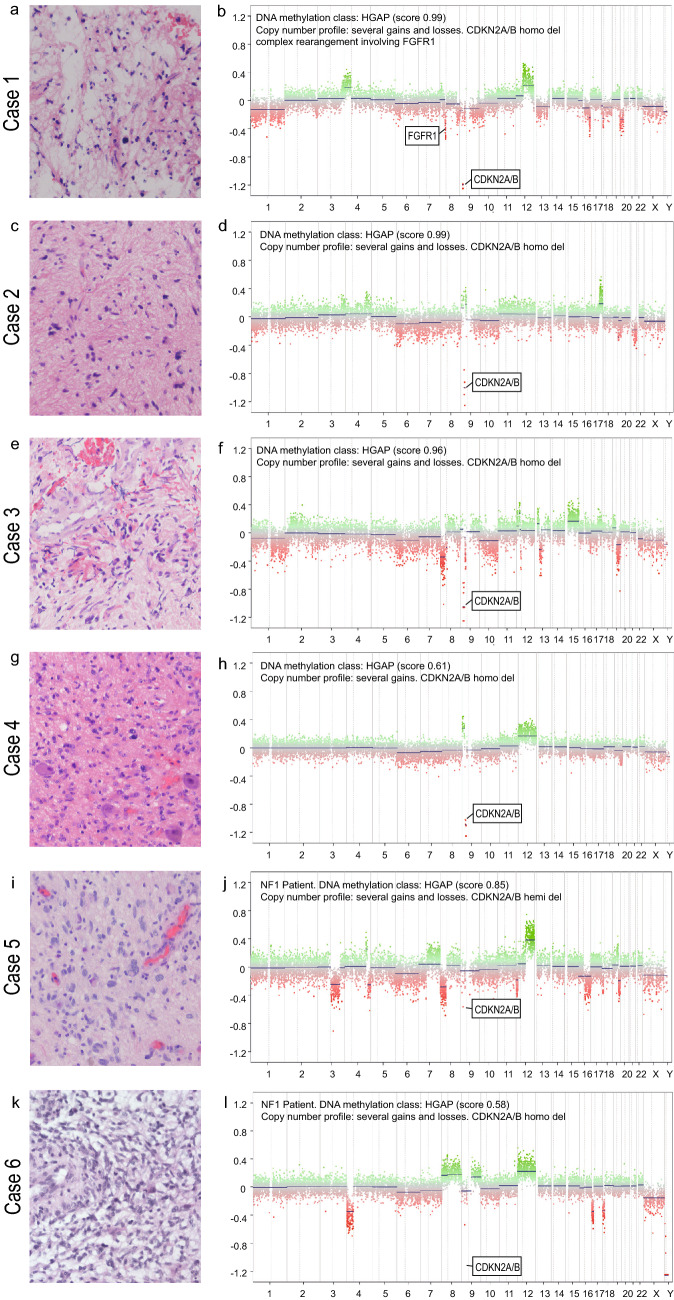
Hematoxylin–eosin stainings and copy number plots. *Patient 1* Hematoxylin–eosin staining revealed low cell density astroglial tumor without marked proliferation. Genome-wide DNA methylation analysis: HGAP (score: 0.99), copy number analysis demonstrated homozygous CDKN2A/B deletion and a complex structural rearrangement of the FGFR1 locus, possibly indicating a gene fusion. The MGMT promoter was non-methylated. *Patient 2* Hematoxylin–eosin staining revealed a moderately cell dense, pleomorphic glial tumor without necrosis, vascular proliferation or marked mitotic activity. The Ki67 proliferation index was focally up to 5% and a nuclear ATRX loss was noted. DNA methylation profiling revealed an HGAP (score: 0.98). Copy number analysis demonstrated homozygous CDKN2A/B deletion. MGMT promoter was non-methylated. *Patient 3* Hematoxylin–eosin staining revealed a low to moderately cell rich, moderately pleomorphic glial tumor with vascular proliferation. Necrosis or mitotic activity was not observed. Ki67 proliferation index was 5–10% and a nuclear ATRX loss was noted. DNA methylation profiling revealed the diagnosis of an HGAP (score: 0.96). Copy number analysis demonstrated homozygous CDKN2A/B deletion. MGMT promoter was non-methylated. *Patient 4* Hematoxylin–eosin staining revealed a moderately cell rich, moderately pleomorphic glial tumor without vascular proliferation, necrosis or mitotic activity. The Ki67 proliferation index was focally up to 5% and ATRX was retained. The DNA methylation profile was not classifiable but showed the highest classifier score for HGAP (score: 0.61). Copy number analysis demonstrated homozygous CDKN2A/B deletion. The MGMT promoter was methylated. The tumor was IDH 1/2, H3F3A and BRAF V600 wild-type. *Patient 5* Hematoxylin–eosin staining a moderately cell rich, moderately pleomorphic glial tumor without vascular proliferation, necrosis or mitotic activity. The Ki67 proliferation index was 5% and nuclear ATRX was lost. Genome-wide DNA methylation analysis confirmed the diagnosis of an HGAP (score: 0.85). Copy number analysis demonstrated homozygous CDKN2A/B deletion. The MGMT promoter was non-methylated. *Patient 6* Hematoxylin–eosin staining revealed a moderately cell rich, malignant glial tumor with vascular proliferation, necrosis, mitotic activity and vascular thrombi. The Ki67 proliferation index was 10% to 15% and ATRX was retained. The DNA methylation profile was not classifiable but showed the highest classifier score for HGAP (score: 0.58). Copy number analysis demonstrated homozygous CDKN2A/B deletion. The MGMT promoter was methylated. The tumor was IDH 1/2, H3F3A and BRAF V600 wild-type. *CDKN2A/B* cyclin-dependent kinase inhibitor 2A/B, *FGFR1* fibroblast growth factor receptor 1

**Fig. 3:**
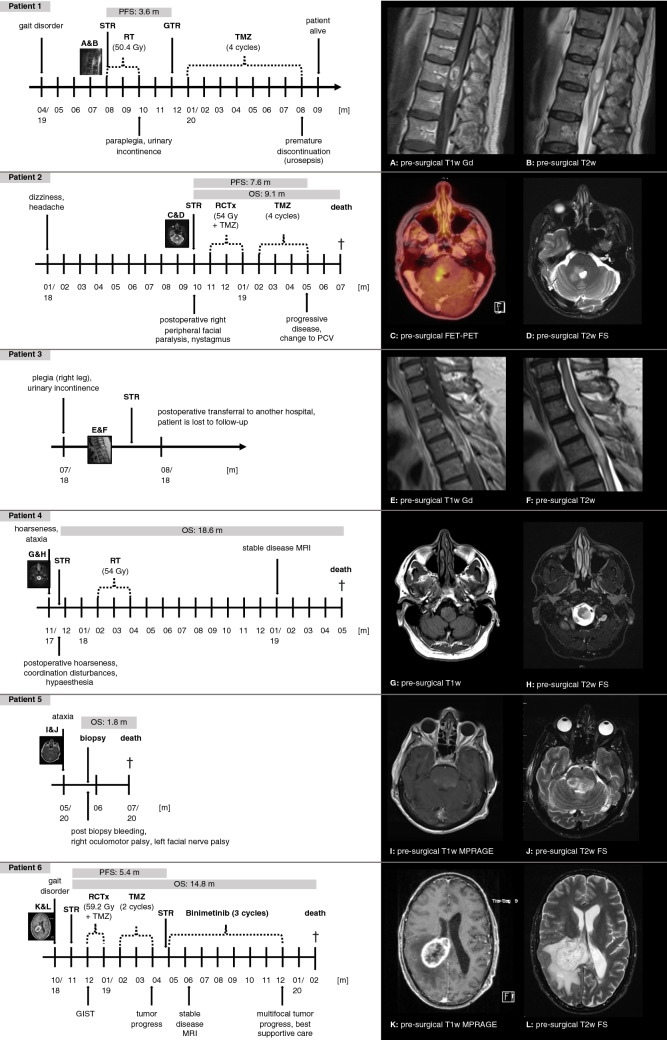
Clinical course of six patients with high-grade astrocytoma with piloid features including pre-operative MRI (**A**–**L**) and O-(2-[^18^F]fluoroethyl)-l-tyrosine positron emission tomography (FET-PET) (**C**). *FS* fat saturation, *Gd* Gadolinium, *Gy* Gray, *MPRAGE* magnetization prepared rapid gradient echo, *MRI* magnetic resonance imaging, *PCV* procarbazine, lomustine, and vincristine, *FET* O-(2-[^18^F]fluoroethyl)-l-tyrosine, *PET* positron emission tomography, *PFS* progression-free survival, *OS* overall survival, *RCTx* radiochemotherapy, *RT* radiotherapy, *STR* subtotal resection, *T* thoracic vertebra, *T1w* T1 weighted image, *T2w* T2 weighted image, *TMZ* temozolomide

We identified six adult patients diagnosed with HGAP between 2017 and 2020. In the same period, a total number of 951 adult patients were treated for newly diagnosed glioma at our institution. Table 1 provides an overview of patient characteristics. Figure 1 demonstrates by t-SNE analysis that all six HGAP cases are grouped with the reference group of HGAP. Figure 2 shows the hematoxylin–eosin stainings and copy number plots. Figure 3 illustrates each patient’s clinical course, including preoperative radiologic imaging.

**Table 1:** Characteristics of six patients with high grade astrocytoma with piloid features

Patient no	Sex/Age	Pathology	Tumor location	Surgery	RT (Gy)	CTx	Postoperative KPS (%)	PFS (m)	OS (m)
1	Male/71	MC HGAP (score 0.99); CDKN2A/B deletion, ATRX loss, FGFR1 complex structural alteration, IDH wild-type, No EGFR amplification, MGMT unmethylated	Spinal cord (T12)	STR → GTR	50.4 Gy	Adjuvant TMZ (4 cycles after re-resection)	60	3.6	Alive at last follow-up, 14.6 months after primary surgery
2	Female/49	MC HGAP (score 0.98); CDKN2A/B deletion, ATRX loss, IDH1 R132H negative, No EGFR amplification, MGMT unmethylated	Pons—right cerebellar peduncle	Stereotactic biopsy	54 Gy	Concomitant TMZ + adjuvant TMZ (4 cycles), followed by PCV protocol	60	7.6	9.1
3	Male/67	MC HGAP (score 0.96); CDKN2A/B deletion, ATRX loss, IDH wild-type, No EGFR amplification, MGMT unmethylated	Spinal cord (T2–T5)	STR	n/a	n/a	n/a	n/a	n/a
4	Male/53	MC HGAP (score 0.61); CDKN2A/B deletion, ATRX retained, IDH wild-type, No EGFR amplification, H3F3A wild-type, BRAF V600 wild-type, MGMT methylated	Brainstem	STR	54 Gy	No	70	n/a	18.6
5	Male/47	MC HGAP (score 0.85); NF1 syndrome, CDKN2A/B deletion, ATRX loss, No EGFR amplification, MGMT unmethylated	Mesencephalon—diencephalon	Stereotactic biopsy	No	No	90	n/a	1.8
6	Male/44	MC HGAP (score 0.58); NF1 syndrome, CDKN2A/B deletion, ATRX retained, IDH1 R132H negative, No EGFR amplification, H3 K27M negative, BRAF V600 wild-type, MGMT methylated	Right parieto-occipital cerebrum	STR → STR	59.2 Gy	Concomitant TMZ + adjuvant TMZ (2 cycles), followed by binimetinib (3 cycles)	70	5.4	14.8

*ATRX* alpha thalassemia/mental retardation syndrome X-linked, *BRAF* B-Raf proto-oncogene, *CDKN2A* cyclin-dependent kinase inhibitor 2A, *CTx* chemotherapy, *FGFR1* fibroblast growth factor receptor 1, *GTR* gross total resection, *Gy* Gray, *H3F3A* H3 histone family member 3A, *IDH* isocitrate dehydrogenase, *KPS* Karnofsky performance score, *m* months, *MC HGAP* methylation class high grade astrocytoma with plioid features, *MGMT* O^6^-methylguanine-DNA-methyltransferase, *n/a* not available, *OS* overall survival, *NF-1* neurofibromatosis type 1, *PCV* procarbazine, lomustine, vincristine, *PFS* progression-free survival, *RT* radiotherapy, *STR* subtotal resection, *T* thoracic vertebra, *TMZ* temozolomide

#### Imaging characteristics

Table 2 summarizes preoperative imaging features of all cases: There seems to be a tendency to rim enhancement with a lack of central enhancement (see Fig. 3a, e, k) resulting in tumor tissue inhomogeneity. In the presented cases the lesions tend to be hypo- to isointense on native T1-weighted (T1w) and primarily hyperintense on T2-weighted (T2w) MRI. Diffusion-weighted imaging (DWI) of three patients revealed no diffusion restriction. In four cases, the tumor margins were sharp, whereas two patients had a diffusely infiltrating tumor. Perilesional edema was present in four cases (see Fig. 3b, d, f, l). There were no radiological signs of cysts, however one case showed signs of necrosis (see Fig. 3k). Preoperative O-(2-[^18^F]fluoroethyl)-l-tyrosine (FET) positron emission tomography (PET) (FET-PET) was carried out in one case (see Fig. 3c), which showed marked tracer uptake. In another case (patient 6), FET-PET was used to detect tumor progression. As more aggressive brain tumor entities usually demonstrate increased FET uptake, FET-PET might be an interesting tool for indicating HGAP.

**Table 2:** MRI features of six pre-surgical HGAP cases

Case no	Size (max. diameter)	MRI T2w	MRI T1w	Enhancement T1w Gd	DWI	Texture		Margins	Surround-ing edema	Cysts/Necrosis	Remarks
						T1w Gd	T2w				
1	30 × 11 mm	Hyperintense	Hypo-/isointense	Tumor rim enhancement with a lack of central enhancement	n/a	Inhomogeneous	Inhomogeneous	Sharp	Yes	No	
2	30 × 13 mm	Hyperintense	Hypo-/isointense	Mild patchy contrast enhancement	No restriction	Inhomogeneous	Inhomogeneous	Diffuse infiltrating	Yes	No	Increased tracer uptake in FET-PET
3	51 × 9 mm	Hyperintense	Hypo-/isointense	Tumor rim enhancement with a lack of central enhancement	n/a	Inhomogeneous	Homogenous	Sharp	Yes	No	
4	18 × 16 mm	Hyperintense	Hypo-/isointense	No Gd applied	n/a	Homogenous	Homogenous	Sharp	No	No	
5	63 × 42 mm	Hyperintense with centrally hypointense parts	Hypo-/isointense	Patchy contrast enhancement	No restriction	Inhomogeneous	Inhomogeneous	Diffuse infiltrating	No	No	Cochlear implant artifact
6	37 × 52 mm	Primarily hyperintense	Hypo-/isointense	Tumor rim enhancement with a lack of central enhancement	No restriction	Inhomogeneous	Inhomogeneous	Sharp	Yes	Necrosis	Increased tracer uptake in FET-PET

*HGAP* high grade astrocytoma with piloid features, *MRI* magnetic resonance imaging, *Gd* gadolinium, *n* native, *FET* O-(2-[^18^F]fluoroethyl)-l-tyrosine, *PET* positron emission tomography

#### Patient 1

In April 2019, a 71-year-old male noticed an unsteady gait and a burning pain in the left leg. Three months after the initial symptoms, a spinal MRI was performed, demonstrating an intramedullary lesion at T12 (30 × 11 mm). This tumor was hypo- to isointense on T1w and inhomogenously hyperintense on T2w with sharply demarcated margins, peripheral contrast enhancement and lack of central enhancement with corresponding myelopathy (see Fig. 3a, b). Three months later (August 2019), the patient underwent subtotal resection via hemilaminectomy at an external institution. The tumor was histologically classified as spinal grade II ependymoma. Adjuvant radiotherapy was performed with a total dose of 50.4 Gy in single doses of 1.8 Gy. Shortly after radiotherapy, the patient presented with paraplegia and urinary incontinence. MRI showed rapid tumor progression with extensive myeloedema, 3.6 months after surgery. The patient was admitted to our institution and gross total resection was performed. Histological examination demonstrated a low cell density astrocytic tumor without marked proliferation (Ki67 proliferation under 1%). ATRX was lost. The finding of a lower grade IDH wild-type glioma prompted genome-wide DNA methylation analysis which revealed an HGAP with classifier score of 0.99. Copy number analysis demonstrated homozygous CDKN2A/B deletion and suggested a complex structural rearrangement of the FGFR1 locus, possibly indicating a gene fusion. The MGMT promoter was unmethylated. KPS after re-resection was 60%. The patient received adjuvant temozolomide chemotherapy for four cycles until discontinuation was necessary due to severe urosepsis. The patient was alive at the time of last follow-up in September 2020 and showed no radiological evidence of disease, 14.6 months after primary surgery.

#### Patient 2

A 49-year-old female presented with dizziness and headaches in January 2018.

FET-PET showed a 30 × 13 mm lesion extending from the pontine tegmentum to the right cerebellar peduncle. The lesion was hypo- to isointense on T1w and hyperintense on T2w sequence. Mild patchy contrast enhancement, a diffuse infiltration of surrounding tissue and perilesional edema were suggestive of a highly malignant brain tumor. DWI indicated no diffusion restriction. FET-PET showed congruent increased tracer uptake (see Fig. 3c, d). Stereotactic biopsy exhibited a moderately cell dense, pleomorphic glial tumor without necrosis, vascular proliferation or marked mitotic activity. The Ki67 proliferation was focally up to 5% and a nuclear ATRX loss was noted. DNA methylation profiling revealed an HGAP with a classifier score of 0.98. Copy number analysis demonstrated a homozygous CDKN2A/B deletion. MGMT promoter was unmethylated. The patient received radiochemotherapy with a single dose of 1.8 Gy to a total dose of 54 Gy and concomitant temozolomide. After completion of radiotherapy, four more cycles of temozolomide were administered. Seven point six months after surgery, the patient reported an increase in number of headache attacks as well as progressive weakness. MRI showed tumor progression in the right cerebellar peduncle. The interdisciplinary tumor board decided to change chemotherapy from temozolomide to PCV (procarbazine, lomustine, and vincristine). However, the patient deteriorated quickly and died 9 months after surgery in July 2019.

#### Patient 3

A 67-year-old male presented with thoracic pain in the dermatome T3. He quickly developed a gait disorder with plegia of the right leg and urinary incontinence. Thoracic spine MRI showed an intramedullary, sharply demarcated, spindle-shaped, and post-contrast rim enhancing inhomogeneous mass (51 × 9 mm) from T2 to T5 with a corresponding myeloedema. The lesion was hypo- to isointense on T1w and hyperintense on T2w sequence (see Fig. 3e, f). The patient subsequently underwent subtotal resection. Postoperatively, neurological symptoms remained unchanged. Histologic evaluation demonstrated a lowly to moderately cell dense, moderately pleomorphic glial tumor with vascular proliferation. Necrosis or mitotic activity was not observed. Ki67 proliferation was 5–10%, IDH 1 R132H immunohistochemistry was negative and nuclear ATRX loss was noted. DNA methylation profiling established the diagnosis of an HGAP with a classifier score of 0.96. Copy number analysis demonstrated a homozygous CDKN2A/B deletion. MGMT promoter status was unmethylated. For personal reasons, the patient received follow-up treatment at another hospital and was lost to outcome follow-up.

#### Patient 4

In November 2017, a 53-year-old male presented with hoarseness and intermittent ataxia. Cerebral MRI showed a sharply demarcated mass (18 × 16 mm) in the dorsal brain stem (see Fig. 3g, h). Only native MRI sequences were available for analysis. In T1w sequences, the tumor appeared homogenously hypointense to isointense. On T2w images, it appeared hyperintense. Subtotal resection was performed. Histologic evaluation demonstrated a moderately cell dense, moderately pleomorphic glial tumor without vascular proliferation, necrosis or mitotic activity. The Ki67 proliferation index was focally up to 5% and ATRX expression was retained. The DNA methylation profile was not classifiable but showed the highest classifier score for HGAP (0.61). Copy number analysis demonstrated homozygous CDKN2A/B deletion. The MGMT promoter was methylated. The tumor was IDH 1/2, H3F3A and BRAF V600 wild-type. Surgery was followed by radiotherapy with a total dose of 54 Gy with single fractions of 1.8 Gy. The last MRI was perfomed 14 months after surgery and showed a stable disease. The patient died 18.6 months after surgery in May 2019.

#### Patient 5

A 47-year-old male patient with known neurofibromatosis type 1 (NF-1) presented in May 2020 with progressive ataxia. The patient had a history of resections of neurofibromas in the head and neck region and a resection of an unclassified right retrobulbar tumor at 7 years of age. MRI showed a diffusely infiltrating, inhomogeneously contrast-enhancing mass (63 × 42 mm) of the mesencephalon and diencephalon (see Fig. 3i, j) and an obstructive hydrocephalus. The tumor presented as hypo- to isointense on T1w and hyperintense with centrally hypointense parts on T2w images. DWI detected no diffusion restriction. A ventriculoperitoneal shunt was placed to treat the hydrocephalus. A stereotactic biopsy was performed shortly after. Histologic evaluation demonstrated a moderately cell dense, moderately pleomorphic glial tumor without vascular proliferation, necrosis or mitotic activity. The Ki67 proliferation index was 5% and nuclear ATRX expression was lost. Genome-wide DNA methylation analysis confirmed the diagnosis of an HGAP with a classifier score of 0.85. Copy number analysis demonstrated homozygous CDKN2A/B deletion. The MGMT promoter was unmethylated. The patient experienced severe post-biopsy bleeding and subsequently presented with a right oculomotor nerve palsy, and a left facial nerve palsy. In the following weeks, imaging demonstrated complete absorption of the hemorrhage and no signs of tumor progression. However, the patient died 1.8 months after surgery.

#### Patient 6

In November 2018, a 44-year-old male with known NF-1 was admitted to our emergency department with general malaise, weakness, nausea, skin pallor and a left spastic hemiplegia. T1w contrast MRI revealed a right parieto-occipital, midline crossing, sharply demarcated, inhomogenously ring-enhancing lesion (37 × 52 mm) with central necrosis (see Fig. 3k). Moreover, several small perifocal enhancing satellites as well as a meningeal enhancement of the right hemisphere were noted. T2w images showed an inhomogeneously, primarily hyperintense tumor surrounded by a large hyperintense edema (see Fig. 3l) and local ependymal infiltration of the right lateral ventricle. DWI detected no diffusion restriction. The patient subsequently received a subtotal resection of the tumor via right parietal craniotomy. The tumor showed histological features of malignancy. DNA methylation was not classifiable but showed highest reminiscence with HGAP (score: 0.58). Molecularly, the tumor presented as IDH 1 R132H negative, ATRX was retained and H3 K27M was negative. The interdisciplinary tumor board suggested using an accelerated hyperfractionated radiochemotherapy protocol with concomitant and adjuvant temozolomide. One week after surgery, the patient presented with progressive anemia and melena and a gastrointestinal stromal tumor (GIST) of the small intestine was diagnosed and treated via laparoscopic tumor resection.

Adjuvant combined concomitant radiochemotherapy was performed. The patient received a total dose of 59.2 Gy delivered in single doses of 1.6 Gy twice a day and concomitant chemotherapy with temozolomide (75 mg/m^2^). After radiotherapy, the patient was treated with two cycles of adjuvant temozolomide until brain PET/MRI revealed a right trigonal tumor progress with an increased uptake in FET-PET, 5.4 months after surgery. Subtotal re-resection was performed. Molecular examination of the tumor showed a truncating NF1 mutation, absence of BRAFV600 hotspot mutation and CDKN2A/B deletion. The interdisciplinary tumor board decided on targeted treatment with the MEK inhibitor binimetinib. The patient received binimetinib 45 mg twice daily for three cycles during which he showed stable disease in imaging. After seven months of binimetinib treatment, the patient was admitted to hospital because of headaches, nausea, emesis, anorexia and intense pruritus. Due to a multifocal tumor relapse in brain MRI and a deterioration of the patient’s general condition, binimetinib was terminated and best supportive care was initiated. The patient died 14.8 months after initial surgery.

## Discussion

Reinhardt and colleagues published the first collection of HGAP cases in 2018. In a reassessment of 102 histologically defined anaplastic pilocytic astrocytomas, DNA methylation analysis revealed a subset of 83 cases of HGAP. HGAP has been described as most common in adults with a median age of 41.5 years [6], slightly younger than the cohort presented here. The tumors most frequently arise in the posterior fossa, with the cerebellum as the most common site. HGAP frequently exhibit molecular alterations of NF1, BRAF, or FGFR1 in combination with CDKN2A/B deletion and/or mutations or loss of ATRX and may have a methylated MGMT promoter. Retrospective data indicate dismal prognosis only slightly better than in IDH wild-type glioblastoma [6].

In 2019, Reinhardt and colleagues reevaluated 86 cerebellar glioblastomas by DNA methylation analysis and found 25 HGAPs [7].

Synoptical clinical information on HGAP patients is scarce: A case series by Gareton et al. analyzed a cohort of 31 pediatric anaplastic pilocytic astrocytoma cases, identifying one case of HGAP. The eight-year-old girl presented with a parietal tumor on the right side. After gross total resection and adjuvant radiochemotherapy, the patient had a PFS of 13.4 months and an OS of 37 months [8]. This case differs from our cases in terms of age, tumor location, surgical intervention, and survival.

Mair et al. presented 46 patients with adult pilocytic astrocytoma (PA). One case was reevaluated at recurrence and DNA methylation-based classification revealed an HGAP. The patients’ clinical course was not described [9].

Two of our patients (patient 5 and 6) had a history of NF-1. In this context, it is noteworthy that patient 5 had a retrobulbar tumor in his childhood. Considering the clinical course, this might have been a case of pilocytic astrocytoma as the optic tract is a predilection site. A relation of NF-1 and development of PA is well established [10], and a causal contiguity of NF-1 and development of anaplastic pilocytic astrocytoma and HGAP has been discussed [6, 11]. The second patient with a known NF-1 (patient 6) is different from our other five cases in terms of tumor location, radiological and histological aspect and therapy approach. The patient’s tumor was, unlike the other tumors, located in the cerebrum, and radiologically resembling a glioblastoma with peripheral contrast enhancement and extensive peritumoral edema. Histologically, there were clear features of malignancy. At first recurrence, the patient received re-resection and targeted treatment with the MEK inhibitor binimetinib. This is, to our knowledge, the first report of targeted treatment in HGAP. The patient clinically benefitted for several months, however, no objective response was recorded.

The frequent MAPK pathway alterations in HGAP are potential therapeutic targets. However, entity-specific evidence for efficacy of targeted treatment is lacking. Specific inhibitors exist for BRAF hotspot mutations and FGFR alterations, while there is a general rationale for use of MEK inhibitors in tumors with upstream MAPK alterations. For example, the MEK inhibitor selumetinib demonstrated activity in BRAF V600E-mutant, KIAA1549:BRAF fusion positive and NF1-mutant (lower grade) gliomas, providing a rationale for its evaluation in HGAP [12]. Similarily, several other MEK inhibitors have shown efficacy in brain metastases of melanoma, demonstrating CNS penetration and intracranial activity [13–15]. Prospective trials evaluating MAPK pathway inhibition in HGAP are urgently needed.

The small number of patients in our case series does not allow stringent characterization of radiologic imaging features but it seems that the lesions tend to be hypo- to isointense on native T1w and hyperintense on T2w MRI. There were no radiological signs of cysts and only one case with necrosis (patient 6). FET-PET was carried out in two cases and showed marked tracer uptake. Patients with a suspected HGAP should receive a standard CNS tumor protocol involving T2w and T1w sequences (e.g. TSE or MPRAGE, FLAIR, DWI, TIRM) and postcontrast T1w sequences (e.g. TSE or MPRAGE). FET-PET should be considered in addition to MRI.

With the exception of case 6, histological analysis did not show signs of increased mitotic activity or necrosis. Despite the absence of histological high-grade malignancy, patients showed poor clinical outcomes. Three patients with brain stem involving tumors died after 1.8, 9.1 and 18.6 months. This may be due to the fact that surgical removal of tumors from this area is difficult. Patient 2 had a tumor relapse after 7.6 months and died only two months later even though she reveiced an extensive treatment including radiation therapy and four cycles of temozolomide. One patient (patient 1) with a spinal tumor diagnosed in August 2019 was alive at the time of the last follow-up in September 2020 and was considered radiolocially free of disease. This patient had received an initial subtotal resection followed by a combined radiochemotherapy and subsequent recurrence. After gross total resection and four adjuvant cylces of temozolomide chemotherapy, he remained tumor free until last follow up. This points out the important role of surgical approaches when possible.

Another issue that has to be discussed is the fact that patients 4 and 6 only showed a DNA methylation score of 0.61 and 0.58. Thus, no definite molecular diagnosis can be made for these tumors. The neurooncologic community will have to agree on a modus operandi on how to handle such unclassifiable tumors in the future.

The HGAP of this series were identified by DNA methylation profiling [2]. In our center we routinely perform DNA methylation profiling of all IDH wild-type diffuse gliomas not showing histological criteria of glioblastoma and of all tumors developing in a tumor syndrome (e.g. NF1). This may explain, why the HGAP of our series presented with fewer features of histological malignancy compared to the cases reported by Reinhardt et al. [7]. And this may further indicate that the number of HGAP at our center may actually be higher than what we report here.

Our data is limited by the small number of cases but indicates that aggressive therapy should probably be performed whenever possible: Therapeutic inhibition of the MAP-kinase pathway shoud be further evaluated prospectively. Methylation profiling is required to establish the diagnosis and should be considered when clinical or histological red flags indicate possible HGAP. More studies are needed to further evaluate the clinical behavior of this tumor entity.

## Data Availability

Data available on request from the authors.

## Abbreviations

ATRX: Alpha thalassemia/mental retardation syndrome X-linked
BRAF: Proto-oncogene B-Raf
CDKN2A/B: Cyclin-dependent kinase inhibitor 2A/B
CNS: Central nervous system
DWI: Diffusion-weighted imaging
FET: O-(2-[^18^F]fluoroethyl)-l-tyrosine
FGFR1: Fibroblast growth factor receptor 1
FLAIR: Fluid-attenuated inversion recovery
GIST: Gastrointestinal stromal tumor
Gy: Gray
H3F3A: H3 histone family member 3A
HGAP: High-grade astrocytoma with piloid features
IDH 1/2: Isocitrate dehydrogenase 1/2
KPS: Karnfosky performance status
MAPK: Mitogen-acitivated protein kinase
MC AAP: Methylation-class anaplastic astrocytoma with piloid features
MEK: Mitogen-activated protein kinase kinase
MGMT: O^6^-methylguanine-DNA-methyltransferase
MPRAGE: Magnetization prepared rapid gradient echo
MRI: Magnetic resonance imaging
NF1: Neurofibromatosis type 1
OS: Overall survival
PA: Pilocytic astrocytoma
PET: Positron emission tomography
PFS: Progression free survival
T: Thoracic
T1w: T1-weighted image
T2w: T2-weighted image
TIRM: Turbo inversion recovery magnitude
TSE: Turbo spin echo
t-SNE: T-distributed stochastic neighbor embedding
WHO: World Health Organization
